# Graduate entry to medicine in Iran

**DOI:** 10.1186/1472-6920-8-47

**Published:** 2008-10-11

**Authors:** Saharnaz Nedjat, Reza Majdzadeh, Arash Rashidian

**Affiliations:** 1 School of public health, Center of Academic and Health Policy (CAHP), Tehran University of Medical Sciences, Tehran, Iran

## Abstract

**Backgrounds:**

In Iran medical students are selected from high school graduates via a very competitive national university entrance exam. New proposals have been seriously considered for admitting students from those with bachelor degrees. We assessed the opinions of different stakeholders on the current situation of admission into medicine in Iran, and their views on positive and negative aspects of admitting graduates into medicine.

**Methods:**

We conducted five focus group discussions and seven in-depth interviews with stakeholders including medical students, science students, university professors of basic sciences, medical education experts, and policy makers. Main themes were identified from the data and analyzed using content analysis approach.

**Results:**

Medical students believed "graduate admission" may lead to a more informed choice of medicine. They thought it could result in admission of students with lower levels of academic aptitude. The science students were in favor of "graduate admission". The education experts and the professors of basic science all mentioned the shortcomings of the current system of admission and considered "graduate admission" as an appropriate opportunity for correcting some of the shortcomings. The policy makers pointed out the potential positive influences of "graduate admission" on strengthening basic science research. They thought, however, that "graduate admission" may result in lengthening the overall duration of medical education, which is already long in Iran (over 7 years). On the whole, the participants thought that "graduate admission" is a step in the right direction for improving quality of medical education.

**Conclusion:**

"Graduate admission" has the potential to correct some of shortcomings of medical education. Unlike other countries where "graduate admission" is used mainly to admit students who are mentally mature, in Iran the main objective seems to be strengthening basic sciences.

## Background

Getting an admission to a public medical school in Iran is challenging and extremely competitive. Admission to public universities, which are tuition free, takes place through national university entrance exams (known as 'Konkoor'). The exams are conducted once a year in five academic streams of 'natural sciences', 'mathematics and physics', 'humanities', 'fine arts', and 'foreign languages'. Each year, about half a million students sit the natural sciences stream of the exam, of which 31,000 are accepted in public universities, and only about 2,700 are admitted into the 52 publicly funded medical schools around the country. These are usually the brightest students (those acquiring the best ranks from the exams); because a career in medical profession is still popular in Iran and that studying medicine is considered 'prestigious'. On the other hand, the future employment status of physicians is not so bright. It is estimated that there are currently 8,000 to 12,000 unemployed physicians in the country [1]. However the definition of 'unemployed' physician is not absolutely clear as many of these physicians may work in fields related to medicine (e.g. research or health services management), or may have changed careers.

Once they enter medical school, the students have to continue their study until they graduate as an MD (Doctor of Medicine) and qualify as general physicians. A tiny fraction of these students pursue career in basic science research, resulting in concerns of not having enough number of basic scientists with deep clinical knowledge.

Students choose medicine based on several social, economical and cultural factors and are not necessarily informed of professional realities of medical jobs or aware of their academic interests. The admission process may result in a situation where applicants choose medicine without having sufficient knowledge of its implications and professional future. In other words, students need to know what they are getting themselves into, and a proper system of assessment is necessary at the time of admission to help identify those who are likely to be able to fulfill the academic and professional requirements of medical careers.

It is expected that when candidates are admitted upon graduation from high school with no voluntary work experience in medicine, the candidates may not be able to evaluate their compatibility with this field of study. Assessing cognitive and meta-cognitive characteristics of the applicants may be helpful in admitting the 'right' candidates into medicine [2-4]. Meta-cognition refers to one's self-awareness of knowledge and ability to comprehend, control and influence one's own cognitive processes [5]. Questionnaires have been designed, standardized, and used in the United Kingdom, Australia and Canada for assessing these characteristics among medical school applicants [6-8]. It seems that it is prudent to consider non-academic criteria in the selection of medical students in Iran, those characteristics which yield 'good physicians'.

Some countries admit medical students at postgraduate levels (after receiving a bachelor's degree or "graduate admission") [9-11]. In Iran, three cohorts of "graduate admission" students enrolled into one medical school in Tehran during 1977 to 1979, but this was discontinued. A few years later, in 1983, a proposal for reestablishing "graduate admission" was presented but the proposal did not gain popularity. In 2003, a similar proposal was considered at the High Council of Programming of the Ministry of Health and Medical Education. The High Council decided that "graduate admission" should be piloted at a limited number of medical schools. These pilots were not realized; for one thing, there were strong views against the proposals among prominent academics and politicians. Since the positive and negative aspects of changing the admission process in Iran had not been rigorously examined, we conducted this study to shed further light on the issue. We examined different stakeholders' perspectives on the current situation of admission into medicine, and their views on positive and negative aspects, if any change was brought forward.

## Methods

### Participants

We approached different groups of stakeholders (medical students, science students, university professors of basic sciences, medical education experts, and policy makers), and assessed their views.

Seven semi-structured in-depth interviews with policy makers and five focus group discussions with students and professors were carried out [12,13]. The interviews were arranged with policy-makers at the Higher Council of Programming of the Ministry of Health and Medical Education; some of them were admitted to medicine with a bachelor's degree in 1977 in Iran. Medical students in the first and sixth year of their studies (10 and 6 students, respectively), fourth-year students of biology, physics, and chemistry (9 students), experts in education (6 faculty members), and professors of basic sciences (4 faculty members) participated in the five focus group discussions. Faculty members and medical students were all from the Tehran University of Medical Sciences (the largest medical university in Iran with over 1,200 academic faculty members). Science students were studying at the Tehran University which is Iran's largest university. Students were selected so that necessary variation in influential parameters such as gender and original place of residence (provincial cities) was achieved as far as possible.

### Interviews and focus group discussions

The research team briefed the participants on the study's objectives before the interviews and focus group discussions began; that it was a research on graduate entry into medicine, and that the researchers were not biased towards this kind of admission. The current process of admission to medical universities, the strengths and weaknesses of the current and "graduate admission" processes, potential solutions to the shortcomings of the new admission process and its implementation were discussed with the participants.

The focus group discussions with the students were recorded and transcribed. Notes from other focus group discussions and the interviews with policy makers were simultaneously taken down by two facilitators or two interviewers respectively. All of those invited to the focus group discussions participated in the study. Only one of the policy-makers was unable to attend the in-depth interview due to an illness. All the transcriptions and notes were reviewed and the main themes were extracted by two individuals independently and coded for further analysis. Content analysis was used for the analysis of the data. The negative instances were also taken into account while analyzing the data [12,13].

## Results

The extracted themes in the analysis of different groups consisted of "reason of choosing medicine", "current status of selecting medical students ", "impact of graduate admission on strengthening basic science and its other positive aspects" and "difficulties of graduate admission into medicine".

### First and Sixth Year Medical Students; the Students of Sciences (potential medical applicants)

Half of the participants implied that the proposed method of "graduate admission" to medicine will decrease the stress of sitting the National University Entrance Examination. Almost everybody agreed that in case the new process is set up, candidates with more mature characters will be admitted to medical schools, and that the students (applicants) will have a better chance of making informed choices. A couple of participants stated that the National Educational Testing Organization can somehow solve the problem of uninformed choices through proper media programs. This is the organization which designs and arranges the universities' national entrance exam and other standardized examinations all over the country. Most thought that "graduate admission" is unlikely to be able in strengthening basic sciences (table 1).

**Table 1:** A sample of students' opinions of the current situation of choosing medicine

*Reason of choosing medicine*
"If someone gains scores high enough to enter the medical field but chooses another field, everybody thinks that he/she is stupid".
"It's important for high schools that their students enter the medical field".
"The medical field is completely different from other fields: the type of studies, its length, the number of courses, its duties. The burden of its work is more than other fields, yet it gives a good feeling."
"Everybody believes that medicine is superior to other fields."
"In other fields, there is no guarantee to reach the doctorate level as advancing to each higher stage requires that the students pass the relevant examinations. But in medicine the students are sure they will at least get their doctorate degree. Whether this degree is useful or not, is another problem."
"Everybody believes that medicine is a distinctive field."

Several participants believed that "graduate admission" to medicine can lead to a fall in the academic aptitude of the students of medicine and medical education; "If the criteria are not exact and rigorous, it is probable that candidates who do not possess real capabilities are admitted to medicine".

All of the medical students believed that students who choose to study medicine are not necessarily aware of their interests in this field. As a result, issues such as the high rank and social prestige of medicine, direct admission into a doctorate program (i.e. MD), the family traditions, and the force of family, friends, and teachers influence the applicants' decisions. Almost all agreed that the candidate's decision is not based on his/her personal interest or insight. Most of the students mentioned the high social prestige of being a physician as one of the main reasons for choosing medicine. In terms of helping people and contributing to the health of their fellow citizens, almost everybody agreed that "although they are discussed in many occasions, they do not practically go further than mere talks and are completely unreal". One participant mentioned "helping people" as one of the main reasons he had chosen medicine, yet he stated that "I don't have that motivation anymore. After all the hard work I ask myself why I should devote my life to others". According to most students, nowadays few students choose medicine out of financial reasons, because the potential income of medical graduates has relatively decreased in recent years.

The continuity of this field to the doctorate degree is one of the reasons candidates choose it. Half of the students implied that studying medicine is associated with a sense of superiority or being distinct from other students (table 1).

The results of our group discussions with current students of sciences (potential applicants for the new admission system) demonstrated similar results. The students of science, on the whole, were in favor of the new process and half of them implied the more informed choice of medicine by bachelor students. In order to strengthen basic sciences, most of the participants believed that fields of science need to receive more attention and research budget. "If students choose medicine after receiving their bachelor's degree, they are interested in medicine and will not return to do research in science". Almost half of the science students believed the project is good and has no disadvantages, while the other half believed "if the project is set up, the basic sciences may be weakened, as the best students in these fields will leave for medicine."

### Professors of Basic Medical Science and Medical Education Experts

None of the participants accepted that the national university entrance exams are a valid and proper way of admitting students to medicine. They all mentioned faults in the current system of admission and considered the new process an appropriate opportunity for correcting it. They believed that the academic merits of top science students were no less than that of medical students. Some of the participants' statements are listed in table 2.

**Table 2:** A sample of the opinions of professors participating in this study towards the current admission

*Reason of choosing medicine*
"Currently, one of the students who had a high rank in the Olympiad of Physics and ranked fourth in the National University Entrance Exam has failed, as he hasn't chosen medicine on his own will."
"Medical students are resources that fall prey to the competition that exists among their parents."
"The student who reasonably and actively decides not to study is more intelligent than one who studies solely out of imitating others. Anyway, the National University Entrance Exam is not a fair and just criterion."
*Current status of selecting medical students*
"Some people indicate that the scientific level of science students is lower than that in medical students, but I don't agree. Their fourth year performance can be evaluated and the admission will be possible with more precision."
*Difficulties of graduate admission into medicine*
"In my opinion, the targets of this project will be achievable through the correction of the educational content; it is not advisable to split the curriculum into two parts."

On the whole, all of the participants were in favor of "graduate admission" to medicine. They considered it an effective step in enabling candidates to make an informed choice of medicine as a major and for improving the current status of the basic sciences. Some of the participants pointed out the necessity of further changes in the educational system.

### Policy-makers (members of the High Council of Educational Planning)

All of the participants (except one person) pointed out the positive influence of the new process in strengthening the basic sciences in the course of medicine (table 3). Five participants (among 7) insisted that changes in the educational content and the curriculum are required for a better learning of basic sciences and improved relevant research. All participants believed that, apart from the new process, a change in the educational content is necessary and should be carried out in advance. One of the participants pointed out the necessity of providing the possibility for students who are not interested in medicine to leave medical studies (while keeping the academic credits that they have earned). He also mentioned that since the number of subjects that require memorization will decrease (i.e. basic science subjects) they will have a better concept on the whole. Most of the participants believed that bachelor degree holders will make a more informed choice at the time of applying to medicine as they are more mentally mature (compared to high school graduates) and have a more comprehensive vision of their choice. Almost half of the participants felt the new approach would be able to admit students with better social and behavioral skills. One of the participants pointed out that it may also be possible to attract students with higher scientific abilities with interest in basic biomedical sciences.

**Table 3:** Some of the policy makers' opinions on the positive and negative aspects of bringing this change in the mode of admission.

*Difficulties of graduate admission into medicine*
"In case the aim is to train the best individuals, prolongation of the course will not create any problem. We want to train the best, so we should spend more time on them."
*Impact of graduate admission on strengthening basic science and its other positive aspects*
"It will be a big problem to have weak basic sciences."
"In my opinion the main motivation behind this task is strengthening the basic sciences."
"This change will make medical students study their subjects from another perspective, and the possibility of introducing innovative methods will rise."

Most participants referred to the older age of the students at the time of graduation as the main disadvantage of the new process. The lack of professors who have positive attitudes towards basic sciences and are aware of their importance was indicated by half of them as a weakness in current medical schools; this may hinder the success of the proposed "graduate admission" in strengthening basic sciences.

Two interviewees pointed out that in case the project is set up only in one university, candidates with lower aptitude will enter that medical faculty through this university. "If only one university accepts this regulation, as people tend to escape from risks, candidates with higher abilities will choose other universities for studying medicine".

Other problems set forth by some of the interviewees were as follows: lack of the necessary abilities in bachelor graduates since bright students choose medicine directly, the probability of a halt in the project in case of any change in higher education management, and the problems of ad hoc planning without defining the details of the process in the long run.

## Discussion

### Stakeholders' perspectives on the current situation of choice of medicine

The results of this study indicate the fact that choosing the field of study by the medical students is not completely based on awareness, and factors such as high esteem, position of physicians in the society, and family (and friends) pressures are also influential. Almost all participants believed that the force of relatives and friends and high prestige of physicians were more influential than other factors. In Australian medical universities, only 52% of the students revealed an obvious interest in medicine; while the force of parents and social esteem were mentioned among other factors. In this study, only 11% of the students stated that they had reasonable knowledge about the field before choosing it [2].

We assessed the motivation behind choosing medicine in a qualitative manner; generally, its quantitative assessment is not an easy task. In these cases, vignettes are usually used for standardization of their responses, because the reasons students state in choosing medicine are strongly influenced by their personal characteristics. In the United Kingdom a questionnaire was designed to investigate the different aspects of choosing the medical field. After a factor analysis, it was clarified that the questionnaire had assessed four factors. These four factors had a strong correlation with the background factors. *Helping people *was related to agreeableness, *Indispensability *was related to a strategic approach to learning, *Respect *was related to a surface approach to learning, and *Science *to openness to experience [14].

### The positive and negative aspects of graduate admission

On the whole, from the stakeholders' point of view, the advantages of the graduate entry into medicine can be divided into three groups: strengthening the basic sciences, a more aware choice of medicine as a major, and finally the possibility of admitting candidates with proper meta-cognitive characteristics.

### Strengthening Basic Sciences

Improvements in the quality of clinical care cannot be achieved without adequate attention to basic sciences. The decline in the number of practitioners who are involved in research and the weakness of basic sciences is not limited to Iran and is globally attended to as a problem [15]. On the other hand, one of the main health policy makers also insisted that there are enough practitioners in the country, and it is time to shift from quantity to quality. Summarizing the group discussions with experts in education and the professors of basic sciences, and concluding from most of the in-depth individual interviews, most stakeholders argued that the graduate admission can have a great effect on strengthening the basic sciences, although some of the medical and bachelor students did not believe so.

All of the professors who took part in focus group discussions pointed to the necessity of effecting changes in the curriculum of basic sciences, complementing the changes brought about by this project. Although strengthening basic sciences is not the ultimate goal of "graduate admission" to medicine in related countries, from the stakeholders' point of view, it seems to be its most important advantage in Iran.

### A More Informed Choice of Medicine as a Major

The results of the current study confirm that the choice of medicine by the candidates of this field is not quite based on awareness and interest in medicine. All of the participants in different groups insisted that if a bachelor's degree is required for admission into medicine, the candidates will possess mental maturity and will choose this field with more awareness. In many creditable medical faculties, a minimum of a bachelor's degree is required. One of the main reasons of this approach is the increased mental maturity which is a result of their previous academic experience, and their higher age at the time of choosing their field of study [9,10,16].

### The Possibility of Admitting Candidates with Meta-Cognitive Characteristics

Choosing students solely on the basis of academic criteria has been laid aside in many countries [2-4]. The necessary meta-cognitive characteristics for medical students include self-portrait, flexibility, communication skills, the power of decision-making and management, logical behavior, honesty and commitment, sympathy, responsiveness, team working abilities, and capability to face stress which can be assessed through tests and interviews [17,18]. Moreover, it has been illustrated in different studies that interviews, rather than different tests, as part of the entrance assessment, can provide additional information and also predict part of the variation in the future performance of the students. Therefore, designing structured interviews is worth spending time and money [7]. According to studies performed all over the world, it is demonstrated that working experience before entering the field of study can be very effective in the choice of profession. That is why voluntary work experience is considered one of the prerequisites for admission to medicine in many creditable medical universities around the world [19].

Admission of bachelor graduates provides the possibility of a more exact inspection of their meta-cognitive characteristics during their bachelor's studies. In the current situation, admission of bachelor students is a valuable opportunity to "improve and adjust" the process of admission. In Australia for example, the non-academic admission based on meta-cognitive characteristics was first performed in one university, and after 10 years of follow-up and observing the relative success of the students, this type of admission was set up all over the country [20]. Therefore, screening candidates for medicine based on these characteristics seems quite reasonable.

Most of the stakeholders pointed out the older age of students as the main setback of the project. It is therefore recommended that a combined MD/PhD degree be offered, or a continuous PhD program be provided so that the assigned public services (and military services in case of men) are performed afterwards. On the other hand, currently the minimum entry age of 23 years has been suggested, even without pre-medicine requirements [21]. This shows that older age can be a strong point of this mode of admission.

The policy makers interviewed in this study were chosen from those based in different universities around the country while being members of the High Council of Educational Planning. The students and faculty were selected from only two universities (one medical and one non-medical university). Hence care should be taken in interpreting the findings. Similar study can be recommended assessing wider views. We also recommend future studies of the effects of the graduate entrée admission when the pilots of this mode of admission are fully in place.

We tried to examine the professional status of 1977–1979 entrée graduates. However, we faced considerable missing data and confounding factors which made it impossible for us to draw any valid conclusions or interpretations.

## Conclusion

Assessment of the change in the stage of admission in universities with similar history has been performed with the intention of admitting students with more maturity, yet the similar suggestion in Iran (besides maturity) is made to improve basic sciences.

There is no doubt that variation in educational styles in different universities can be the start for improving such activities, and should this principle be accepted, admitting bachelor graduates into medicine can be established in voluntary universities.

In any case, if this project is to be set up, two task forces should be formed to correct the admission process, and to change the educational content. Furthermore, monitoring and evaluation of all of the executive stages of the project to introduce necessary alterations is also quite vital and necessary. The effect of this change on professional success should be evaluated in future to have valid inference on the results of this study.

## Competing interests

We conducted this study in response to the TUMS's top management as they were considering whether to open a new "graduate admission" route for studying medicine at the TUMS. The decision to submit the paper and its content has been solely with the authors. The findings of the study, and the TUMS's subsequent decision on whether or not to start the "graduate admission" have no effect on the authors' academic status or salary.

## Authors' contributions

SN and RM participated in the design, data collection, analysis and writing the manuscript. AR participated in the design and writing the manuscript. All the authors approved the final manuscript.

## Pre-publication history

The pre-publication history for this paper can be accessed here:


